# Heterologous Display of *Chlamydia trachomatis* PmpD Passenger at the Surface of *Salmonella* OMVs

**DOI:** 10.3390/membranes13040366

**Published:** 2023-03-23

**Authors:** Dung T. Huynh, Wouter S. P. Jong, Manon A. H. Oudejans, H. Bart van den Berg van Saparoea, Joen Luirink, Peter van Ulsen

**Affiliations:** 1 Abera Bioscience AB, 750 26 Uppsala, Sweden; 2 Department of Molecular Microbiology, Amsterdam Institute of Molecular and Life Sciences (AIMMS), Vrije Universiteit, 1081 HV Amsterdam, The Netherlands

**Keywords:** *C. trachomatis*, PmpD, autotransporter, OM, OMVs, mucosal vaccine

## Abstract

*Chlamydia trachomatis* is the bacterial pathogen that causes most cases of sexually transmitted diseases annually. To combat the global spread of asymptomatic infection, development of effective (mucosal) vaccines that offer both systemic and local immune responses is considered a high priority. In this study, we explored the expression of *C. trachomatis* full-length (FL) PmpD, as well as truncated PmpD passenger constructs fused to a “display” autotransporter (AT) hemoglobin protease (HbpD) and studied their inclusion into outer membrane vesicles (OMVs) of *Escherichia coli* and *Salmonella* Typhimurium. OMVs are considered safe vaccine vectors well-suited for mucosal delivery. By using *E. coli* AT HbpD-fusions of chimeric constructs we improved surface display and successfully generated *Salmonella* OMVs decorated with a secreted and immunogenic PmpD passenger fragment (aa68-629) to 13% of the total protein content. Next, we investigated whether a similar chimeric surface display strategy could be applied to other AT antigens, i.e., secreted fragments of Prn (aa35-350) of *Bordetella pertussis* and VacA (aa65-377) of *Helicobacter pylori*. The data provided information on the complexity of heterologous expression of AT antigens at the OMV surface and suggested that optimal expression strategies should be developed on an antigen-to-antigen basis.

## 1. Introduction

*C. trachomatis* is the most common sexually transmitted pathogen worldwide. Its complex, partly intracellular lifecycle and ability to enter a “persistence phase” when meeting harmful conditions hampers the treatment with conventional antibiotics [1]. Importantly, chlamydial infections often remain asymptomatic, which contributes to its transmission in a sexually active population. Consequently, development of a (mucosal) vaccine that offers protection against transmission and/or chlamydial disease is considered a high priority [2]. Research has focused on both whole-cell-based and subunit-based vaccine formulations but has not yet led to a licensed vaccine [3].

*C. trachomatis* major outer membrane protein (MOMP) is the best studied antigen included in subunit vaccines. It is used in more than half of the published preclinical studies [3] and CTH522, a recombinant non-native MOMP-derivative, has recently entered into clinical phase [4]. The porin is strongly immunogenic and contains various linear and conformational T- and B-cell epitopes [5]. Unfortunately, however, recombinant MOMP in its native conformation is difficult to produce. Other challenges include the partial and serovar-specific immunity it elicits, as well as the variable protection against (re)infection shown in preclinical studies [6].

Despite the ongoing research on MOMP-containing subunit vaccines, alternative chlamydial outer membrane (OM) antigens such as OmcB, Pgp3 and polymorphic membrane proteins (Pmps) are also considered. In particular, the Pmps are attractive antigens as they are surface-exposed, relatively conserved, immunogenic and important for chlamydial pathogenesis [7]. Pmps belong to the large family of monomeric autotransporter (AT) virulence factors [8]. AT proteins comprise three functional domains: an N-terminal signal peptide to direct translocation across the inner membrane (IM) via the Sec-translocon, a central effector domain known as the passenger that is either secreted or surface localized and a C-terminal β-barrel domain. The latter assists in the translocation of the passenger across the OM in an intricate mechanism that also involves the Bam complex [9]. The secreted passengers of ATs in most cases fold into a stable β-helical stem structure and their folding at the cell surface is thought to drive OM translocation. Chlamydial Pmps were predicted to share the typical AT domain structure including the β-helical fold of the passenger [10]. *C. trachomatis* genomes encode up to nine different Pmp proteins, PmpA–H, of which PmpD is the best studied. PmpD functions as an adhesin at an early stage in the host-cell interaction and was shown to be a target of neutralizing antibodies [11]. Once arrived at the cell surface, the PmpD passenger undergoes oligomerization but also further proteolytic processing. This cleavage generates secreted fragments, which may play additional roles in virulence [8,12]. Several preclinical studies have highlighted *C. trachomatis* full-length (FL) PmpD and the processed PmpD passenger fragments as promising antigens suitable for mucosal application [13,14,15]. However, only few mucosal vaccines are at the moment licensed for human use, which is mainly due to the lack of safe and effective mucosal adjuvants and antigen delivery systems [16].

Bacterial outer membrane vesicles (OMVs) have emerged as promising immunomodulating vaccine vectors well-suited for mucosal delivery of recombinant antigens [17,18]. OMVs are nanoparticles that naturally shed from the surface of Gram-negative bacteria during growth. Importantly, they present surface components such as lipopolysaccharide (LPS), lipoproteins, flagellin and other pathogen-associated molecular patterns (PAMPs) that could serve as potent intrinsic adjuvants [19]. Furthermore, several OMV-derived vaccines preventing meningococcal disease have been licensed for use in humans [20]. Our laboratory has developed an OMV vaccine platform based on an attenuated *Salmonella* Typhimurium strain that has been genetically modified to make it hypervesiculating and to include LPS with a reduced reactogenicity [21]. For recombinant antigen display at the OMV surface, we developed a “display” platform using a non-secreted variant of the *E. coli* AT hemoglobin protease (HbpD), in which surface-exposed parts were replaced by antigen fragments [22]. Of note, there is a limit to the structural complexity of the antigens that can be introduced into the HbpD carrier [23]. To address this issue, we explored covalent coupling of purified recombinant antigens to surface-exposed HbpD using the SpyTag/SpyCatcher protein ligation technology [24]. Large and complex antigens could indeed be presented at the surface of OMVs [21,25,26,27], but the coupling of separately purified antigens complicates the vaccine production process and makes it more costly.

In the current study, we investigated the inclusion of both full-length (FL) PmpD and HbpD-fused truncated PmpD passenger fragments in OMVs. Our results show that *Salmonella* OMVs can be efficiently decorated with truncated PmpD passenger (aa68-629). However, when we applied the same informed fusion approach to secreted fragments of two other AT passengers, i.e., pertactin (Prn; aa35-350) of *Bordetella pertussis* and vacuolating cytotoxin A (VacA; aa65-377) of *Helicobacter pylori*, both showed poor surface display in OMVs. It suggests that an optimal strategy requires an antigen-for-antigen approach and may require alternatives such as the SpyTag/SpyCatcher coupling technology.

## 2. Materials and Methods

### 2.1. Strains and Growth Conditions

*E. coli* DH5α and BL21(DE3) (Novagen, Germany) strains were used for cloning and protein expression, respectively. Hypervesiculating *E. coli* BL21(DE3)omp8 and *Salmonella* Typhimurium SL3261 Δ*tolRA* Δ*msbB* were used for the isolation of OMVs [21,28]. *E. coli* cells were cultured at 37 °C in lysogeny broth (LB; 10 g/L tryptone, 10 g/L NaCl and 5 g/L yeast) containing 0.2% glucose. *Salmonella* cells were grown at 30 °C in TYMC medium (10 g/L tryptone, 5 g/L yeast, 2 mM MgCl_2_ and 2 mM CaCl_2_). Chloramphenicol (30 μg/mL) and/or kanamycin (25 μg/mL), both from Sigma-Aldrich (Saint Louis, MO, USA), were added when appropriate.

### 2.2. Reagents and Sera

Restriction enzymes were obtained from New England Biolabs (Ipswitch, MA, Maine). Lumi-light substrate was purchased from Roche (Mannheim, Germany). Phusion High Fidelity DNA polymerase and Pierce Silver stain kit were from Thermo Fischer Scientific (Waltham, MA, USA). Proteinase K and phenylmethanesulfonyl fluoride (PMSF) were from Sigma-Aldrich (Saint Louis, MO, USA). In-Fusion HD plus kit for seamless DNA cloning was obtained from Takara Bio (Mountain View, CA, USA). Coomassie Blue G250 was from Biorad (Oxford, UK). N-terminal amino acid sequence was determined by Edman degradation performed by Alphalyse A/S (Odense, Denmark).

Polyclonal rabbit antiserum against periplasmic protease DegP was a gift of Jon Beckwith (Harvard Medical School, USA) [29]. Polyclonal antisera against the *E. coli* Hbp passenger (J40) and β-domain (SN477) have been described previously [30,31]. Antisera against the *E. coli* Bam complex, involved in OMP biogenesis, the *C. trachomatis* adhesin-like passenger PmpD and the *E. coli* OM-based lipoprotein LpoB were kindly provided by Tanneke den Blaauwen (University of Amsterdam, Amsterdam, The Netherlands), Harlan Caldwell (National Institutes of Health, USA) and Waldemar Vollmer (Newcastle University, Newcastle upon Tyne, UK), respectively [11,32,33]. Goat anti-rabbit IgG conjugated to horseradish peroxidase was ordered from Rockland (Limerick, PA, USA).

### 2.3. Plasmid Construction

All PCR primers used for plasmid construction (Table S1) and amino acid sequences of the expressed proteins are listed in the supplementary file.

The plasmid pLemo-PmpD (FL) was constructed to express PmpD (FL), which is a full-length version of *C. trachomatis* serovar L2 (strain 434/Bu/ATCC VR-902B) PmpD carrying an *E. coli* Hbp AT signal peptide, 8 residues of its mature, processed part after passage of the cytoplasmic membrane, an HA-tag, and an *E. coli* Bam recognition motif (RYSF) at its C-terminus [34]. To avoid erroneous disulfide bond formation, twenty-four cysteines encoded in PmpD (FL) were replaced by serines. Two overlapping and *E. coli* codon-optimized DNA sequences encoding PmpD (FL) were synthesized by Geneart/Thermo Fisher Scientific, assembled and ligated into the *Sal*I and *Bam*HI sites of pLemo [35] using In-Fusion cloning to yield pLemo-PmpD (FL), controlled by the *rhaBAD* promoter [36].

All other expression plasmids were derivatives of vector pEH3, with constructs under control of an IPTG-inducible *lacUV5* promoter [37], of which pEH3-HbpΔβcleavage has been described previously [23].

A DNA fragment encoding PmpD (aa68-698) was amplified by PCR using pLemo-PmpD (FL) as a template and primers 1 and 2. The resulting product was ligated into the *Sac*I and *BamH*I sites of pEH3-HbpD(Δd1)-SpC [24] to generate pEH3-HbpD(Δd1)-PmpD (aa68-698). Plasmids pEH3-HbpD(Δd1)-Prn (aa35-574) and pEH3-HbpD(Δd1)-VacA (aa65-493) were constructed using a similar strategy using genomic DNA of *B. pertussis* (strain Tohama I) [38] and *H. pylori* (strain J99) [39] as templates, respectively.

To obtain plasmids expressing fusions of the heterologous passengers to HbpD(840), two partly overlapping PCR products were generated encoding the heterologous passengers and a segment of the HbpD (aa840-921), respectively. In all cases, two PCR products were combined and ligated into the *Sac*I and *Kpn*I sites of pEH3-HbpD(Δd1)-PmpD (aa68-698) using In-Fusion cloning. To create pEH3-HbpD(840)-PmpD (aa68-629), the first overlapping fragment was generated by PCR using primers 7 and 8 and pEH3-HbpD(Δd1)-PmpD (aa68-698) as a template. The second fragment was generated using primers 9 and 10 and pEH3-HbpD(Δd1)-PmpD (aa68-698) as a template. A similar strategy was used to construct pEH3-HbpD(840)-Prn (aa35-350) and pEH3-HbpD(840)-VacA (aa65-377) using primers listed in Table S1 and pEH3 derivatives as templates.

### 2.4. Recombinant Protein Expression

Cultures of *E. coli* BL21(DE3) and BL21(DE3)omp8 strains harboring pLemo-PmpD (FL) were grown to an OD_600_ of 0.4–0.5, induced for expression by addition of 8 mM L-rhamnose and then further incubated for 2 h [40]. Cultures of *E. coli* BL21(DE3) and BL21(DE3)omp8 cells harboring pEH3-derivatives were grown until an OD_600_ of 0.4–0.5 and then induced with 0.1 mM IPTG for 2 h for expression of the HbpD-fusions [23]. Cultures of *Salmonella* SL3261 Δ*tolRA* Δ*msbB* cells harboring pEH3-derivatives were first grown to an OD_600_ of 1.0–2.0, diluted to an OD_600_ of 0.02, after which growth was continued overnight in the presence of 0.1 mM IPTG to induce expression of the HbpD-fusions [24]. Culture samples were subjected to centrifugation (12,000× *g*, 1 min, RT) to separate the bacterial cells from the medium. The cell pellets were resuspended in PBS and then mixed with SDS-PAGE loading buffer (f.c. 125 mM Tris-HCl, pH 6.8, 4% SDS, 20% glycerol, 0.02% bromophenol blue, 100 mM DTT), heated at 97 °C for 10 min and analyzed on 10% SDS-PAGE gels. Gels were further subjected to Coomassie Blue G250- and silver-staining or immunoblotting. Coomassie- or silver-stained gels were imaged using a Molecular Imager GS800 Calibrated Densitometer (Biorad). The percentage of each recombinant chimera over total OMV proteins was estimated by densitometry using ImageJ (downloaded from https://imagej.net/ij/index.html; accessed on 20 April 2022) [41]. Immunoblotting images were captured using the Amersham Imager 600 (GE Healthcare, MA, USA).

### 2.5. Isolation of Cell Envelopes and Outer Membrane Fractions

Cell envelopes (CEs) were collected using a subcellular fractionation procedure as described previously [40]. In short, ~200 OD_600_ units of BL21(DE3)omp8 cells expressing PmpD (FL) were resuspended in lysis buffer (100 mM NaCl, 1 mM EDTA, 50 mM Tris HCl, pH 8.0) in the presence of protease inhibitor (Roche, Mannheim, Germany). The suspension was kept in ice for 15 min after which the cells were lysed by two passages through a One-shot cell disruptor (Constant Systems LtD, Daventry, UK) at 1.9 kbar. Whole-cell lysates were centrifuged at 6000× *g*, 10 min, 4 °C to remove debris and unbroken cells and the resulting supernatant was applied to ultracentrifugation (293,000× *g*, 60 min, 4 °C) to sediment CEs.

OMs were subsequently purified by sucrose gradient ultracentrifugation using a procedure adapted from Ruiz et al. [42]. Briefly, CEs collected above were resuspended in 8 mL Tris-B buffer (10 mM Tris-HCl, pH 8.0), containing 20% (*w*/*w*) sucrose and 1 mM PMSF. The suspension was layered onto a two-step sucrose gradient (top: 4 mL Tris-B buffer containing 40% (*w*/*w*) sucrose; bottom: 1 mL Tris-B buffer containing 65% (*w*/*w*) sucrose). After ultracentrifugation (87,000× *g*, 20 h, 4 °C) in a Beckman SW28-Ti rotor with slow acceleration and no braking, a ~0.5 mL volume containing OMs was harvested from the 40%/65% interface by puncturing a side of the tube with a syringe needle. The harvested fraction was diluted three times with washing buffer (20 mM Tris-HCl, pH 8.0) and subjected to ultracentrifugation (18,000× *g*, 30 min, 4 °C) to collect OMs. The pellet was resuspended in TAE-sucrose buffer (50 mM TEA, 250 mM sucrose) and OM material was stored at −20 °C until used.

### 2.6. OMVs Isolation

Cultures of *E. coli* BL21(DE3)omp8 or *Salmonella* SL3261 Δ*tolRA* Δ*msbB* were subjected to centrifugation (6000× *g*, 10 min, 4 °C) to separate the cells and the medium [24,43]. The culture medium was passed through 0.45-μm-pore-size filters (Millipore) and ultracentrifuged (235,000× *g*, 60 min, 4 °C) to sediment OMVs. The OMVs were finally resuspended in PBS at a concentration of 1 OD/µL. An amount of 1 OD unit of OMVs was derived from 1 OD_600_ unit of *E. coli* or *Salmonella* cells.

### 2.7. Proteinase K Accessibility Assay

The proteinase K assay was adapted from a previous study [22]. *E. coli* or *Salmonella* OMVs were resuspended in reaction buffer (50 mM Tris-HCl, pH 7.4, 1 mM CaCl_2_). Where appropriate, OMVs were lysed by incubation with 0.02% (*v*/*v*) Triton X-100 for 15 min in ice. Intact and lysed OMVs were incubated for 30 min at 37 °C in the presence of 100 μg/mL proteinase K (ProK). Intact OMVs incubated under identical conditions in reaction buffer without ProK served as untreated control. Reactions were stopped by addition of 4 mM PMSF, after which all samples were precipitated with trichloroacetic acid and analyzed by SDS-PAGE and Coomassie- or silver-staining and immunoblotting.

## 3. Results

### 3.1. Expression and Localization of Full-Length PmpD (FL)

**Figure 1:**
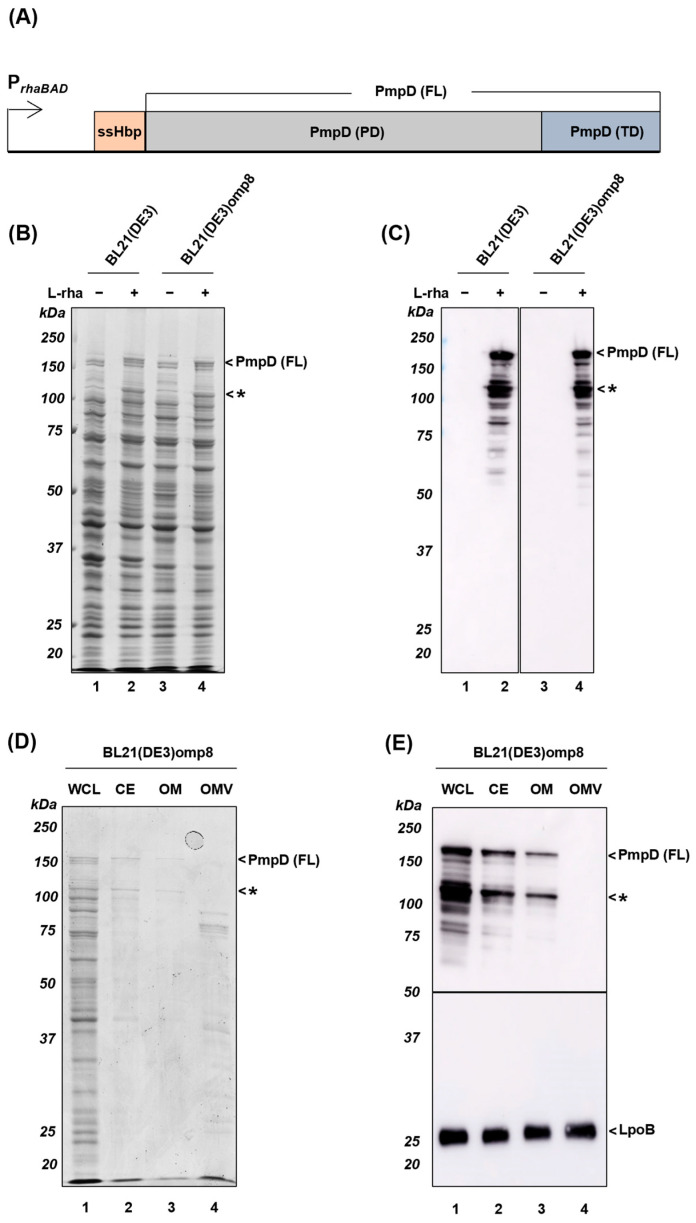
Expression of *C. trachomatis* PmpD (FL) in different *E. coli* strains. (**A**) Schematic representation of the pLemo-PmpD (FL) construct. *E. coli* Hbp signal sequence (ssHbp) fused to *C. trachomatis* PmpD (FL) that consists of passenger and β-barrel domain under control of the *rhaBAD* promoter. (**B**,**C**) Whole-cell lysates of *E. coli* BL21(DE3) and BL21(DE3)omp8 cells induced with (+) or without (−) 8 mM L-rhamnose for 2 h for expression of PmpD (FL) were analyzed by SDS-PAGE, followed by Coomassie staining (**B**) or immunoblotting using PmpD antiserum (**C**). (**D**,**E**) A cellular subfractionation of BL21(DE3)omp8 cells expressing PmpD (FL) resulted in whole-cell lysate (WCL), cell envelope (CE) and outer membrane (OM) fractions. BL21(DE3)omp8 outer membrane vesicles (OMV) were collected from the filtered culture supernatant. Samples derived from 0.05 OD_600_ units of WCL, CE, OM, as well as the equivalent 4 OD_600_ units of *E. coli* OMVs were analyzed by SDS-PAGE followed by Coomassie staining (**D**) and immunoblotting against PmpD or LpoB antisera as indicated. In the panels (**B**–**E**), the proteolytic product of PmpD (FL) of ~120 kDa was annotated (*). MW markers (in kDa) are indicated at the left and identified protein bands at the right side of panels.

In absence of a solved structure of *C. trachomatis* PmpD (FL), we used Alphafold2 [44] to construct models of PmpD (FL) that guided the construct design (Figure S1). As expected, the model showed an N-terminal right-handed β-helix and a C-terminal 12-stranded β-barrel, both with a high similarity to solved structures of ATs [10]. Considering the relatively autonomous and conserved mechanism of AT secretion, we initially expressed PmpD (FL) in *E. coli* in an attempt to obtain OMVs displaying the antigen in its native conformation. *C. trachomatis* PmpD (FL) was placed under control of the L-rhamnose inducible *rhaBAD* promoter (Figure 1A) that allows for tunable expression of recombinant proteins in *E.coli* [35]. The native signal peptide of PmpD was replaced by the *E. coli* Hbp signal peptide for optimal engagement of the Sec translocon in the *E. coli* IM. In addition, twenty-four cysteine residues present in the chlamydial PmpD were replaced by serine residues to avoid disulfide bond formation in the *E. coli* periplasm, because this is known to hinder AT translocation across the OM [45]. Finally, the four C-terminal residues of PmpD (RLIF) were replaced by those of *E. coli* AT Hbp (RYSF) to facilitate efficient recognition by the *E. coli* Bam complex [34]. This motif of four residues is also known as the β-signal and promotes interactions with the Bam complex in a species-specific fashion [46,47]. The optimized PmpD (FL) was expressed in *E. coli* BL21(DE3) and whole-cell lysates from induced and non-induced cells were analyzed by SDS-PAGE and Coomassie staining or immunoblotting using PmpD antiserum (Figure 1B,C). Induction yielded a ~160 kDa product that was readily detectable (Figure 1B, lane 2 and Figure 1C, lane 2) indicating efficient expression. Moreover, a prominent ~120 kDa product (*) was identified on Coomassie-stained gels (Figure 1B, lane 2), presumably corresponding to a proteolytic product [8].

To characterize the proteolytic product, the ~120 kDa band (*) (Figure 1B, lane 2) was subjected to N-terminal amino acid sequencing [48]. The resulting sequence, GTVNN, corresponded to the first five amino acids of the mature protein, present directly downstream of Hbp signal peptide after it is cleaved from the PmpD (FL) precursor by signal peptidase upon passage of the *E. coli* IM (see supplementary file). Hence, the proteolytic product represents a ~120 kDa N-terminal fragment of the PmpD passenger and resembles a similar-sized, cell-associated proteolytic product found upon expression of native PmpD in *C. trachomatis*, suggesting similar processing of our PmpD (FL) construct in *E. coli* [49].

Next, we investigated whether PmpD (FL) is located at the surface of OMVs, which is necessary to enable vesicle-based vaccine formulations. As expression host we used *E. coli* BL21(DE3)omp8 that lacks four major OMPs (OmpA, OmpF, OmpC, LamB) and, consequently, shows a hypervesiculating phenotype [28]. Expression of PmpD (FL) in BL21(DE3)omp8 revealed similar levels of the ~160 and ~120 kDa PmpD products in whole cell lysate samples as detected in BL21(DE3) (Figure 1B, lane 4 and Figure 1C, lane 4). Subsequently, crude CE and OM fractions were isolated from the cells expressing PmpD (FL) and analyzed in parallel to the cell lysates. Coomassie staining (Figure 1D) and immunoblotting using PmpD antiserum (Figure 1E) showed that the ~120 and ~160 kDa species could be recovered from both fractions, consistent with correct biogenesis of PmpD (FL). To monitor recruitment to OMVs, cell-free supernatants of culture samples were subjected to ultracentrifugation and the OMV-containing pellet was analyzed for the presence of PmpD (FL). However, PmpD (FL) could not be detected in this fraction by Coomassie staining (Figure 1D, lane 4) nor by immunoblotting (Figure 1E, lane 4). In contrast, the OM-based lipoprotein LpoB was clearly detected, which verifies the OMV isolation procedure and the loading of similar amounts of OM and OMV material, respectively (Figure 1E, c.f. lanes 3 and 4) [50]. Taken together, PmpD (FL) was successfully targeted to the OM of *E. coli* but was not included in OMVs.

### 3.2. Display of Antigenic Passengers upon Fusion to Hbp in E. coli

Since PmpD (FL) was not detected in OMVs, we then tried to display the immunogenic passenger part that is secreted in *C. trachomatis* on the surface of *E. coli* as part of a chimeric AT construct. Previously, we have used HbpD(Δd1), an engineered version of *E. coli* AT Hbp, for antigen display [22]. In this construct, antigens are positioned on top of the β-helical core of the Hbp passenger through genetic replacement of its native domain 1 (d1) (Figure S2A). Given the predicted structural similarities of the Hbp and PmpD passengers, we considered such a fusion a promising display strategy, despite the considerable size and folding complexity of the truncated PmpD (Figure S2B). To explore the generic applicability of this fusion strategy, we also tested display of truncates of *B. pertussis* Prn and *H. pylori* VacA, two alternative AT virulence factors with β-helical structures (Figure S2B).

**Figure 2:**
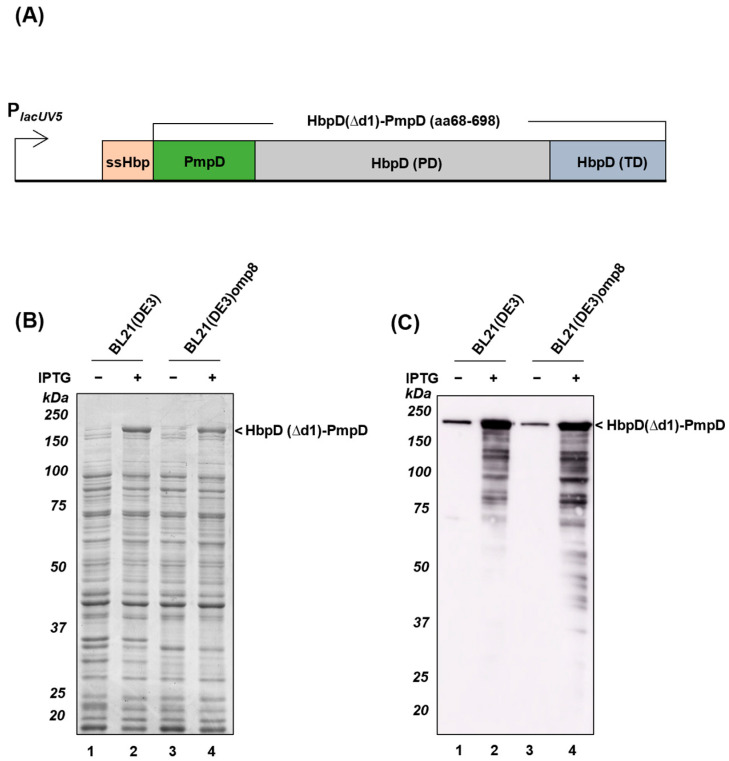
Expression of HbpD(Δd1)-PmpD in different *E. coli* strains. (**A**) Schematic representation of the HbpD(Δd1)-PmpD (aa68-698) construct in which truncated PmpD (aa68-698) was fused to the HbpD(Δd1)-based platform and expressed under control of the *lacUV5* promoter. (**B**,**C**) BL21(DE3) and BL21(DE3)omp8 cells were induced for HbpD(Δd1)-PmpD expression with (+) or without (−) 0.1 mM IPTG for 2 h and whole cells were analyzed by SDS-PAGE/Coomassie staining (**B**) and immunoblotting using antiserum against PmpD (**C**). MW markers (in kDa) are indicated at the left and identified protein bands at the right side of panels.

We constructed plasmids pEH3-HbpD(Δd1)-PmpD (aa68-698), pEH3-HbpD(Δd1)-Prn (aa35-574) and pEH3-HbpD(Δd1)-VacA (aa65-493) to produce the respective fusions to HbpD(Δd1) (Figure 2A, Figures S3A and S4A). Expression was tested in both *E. coli* BL21(DE3) and BL21(DE3)omp8 and cell lysate samples were analyzed by SDS-PAGE and Coomassie staining (Figure 2B, Figures S3B and S4B). Expression of the HbpD(Δd1)-PmpD, -Prn and -VacA fusions was detected in both strains, with bands appearing upon induction at ~190 kDa (Figure 2B), ~180 kDa (Figure S3B) and ~170 kDa (Figure S4B), which corresponds to their calculated molecular masses. The identity of HbpD(Δd1)-PmpD could be confirmed by immunoblotting using antiserum against PmpD (Figure 2C), whereas the other two HbpD(Δd1)-fusions were detected using Hbp-directed antiserum (Figures S3C and S4C). Expression levels were variable with HbpD(Δd1)-VacA being less well detectable (c.f. Figure 2B, lane 4 and Figure S4, lane 4), while expression of this construct also strongly influenced the cell growth (data not shown).

**Figure 3:**
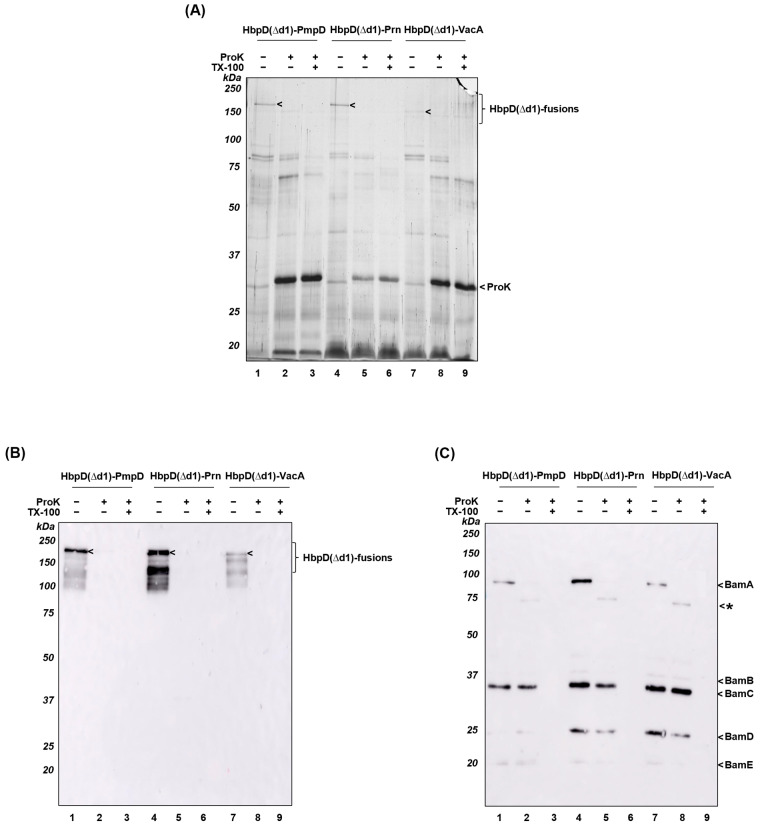
Display of HbpD(Δd1)-PmpD, HbpD(Δd1)-Prn and HbpD(Δd1)-VacA at the surface of *E. coli* OMVs. Equivalent amounts of BL21(DE3)omp8 OMVs expressing HbpD(Δd1)-PmpD; HbpD(Δd1)-Prn and HbpD(Δd1)-VacA were used for a proteinase K (ProK) accessibility assay. Samples of untreated intact OMVs, intact OMVs treated with ProK and lysed OMVs, treated with TritonX-100 (TX-100) and incubated with ProK were analyzed by SDS-PAGE followed by silver staining (**A**) or immunoblotting using Hbp antiserum (**B**) or antiserum recognizing the *E. coli* Bam complex (**C**). In the panel (**C**) bands representing truncated BamA were indicated (*). MW markers (in kDa) are indicated at the left and identified protein bands at the right side of panels.

In the next step, we tested whether HbpD(Δd1)-PmpD, -Prn and -VacA were included in OMVs. OMVs were isolated from the culture medium of BL21(DE3)omp8 cells and analyzed by SDS-PAGE followed by silver staining (Figure 3A) and immunoblotting using Hbp antiserum (Figure 3B). All three fusions appeared to be present in OMVs (Figure 3A,B, lanes 1, 4, 7), albeit with a relatively modest density for HbpD(Δd1)-VacA (Figure 3A,B, lane 7). Of note, due to the use of BL21(DE3)omp8 as the OMV production host, typical porin bands (OmpA/F/C) are absent from the OMV protein profiles (Figure 3A). To assess surface exposure of the heterologous passenger domains, the OMVs were treated with ProK to digest external proteins. Clearly, the HbpD(Δd1)-fusions were degraded (Figure 3A,B, lanes 2, 5, 8). As a control for OMV integrity, we analyzed the accessibility of the partially exposed BamA subunit of the Bam complex (Figure 3C). These control experiments showed that BamA was cleaved by ProK, whereas the intracellular BamB-E components of the complex remained intact (Figure 3C, lanes 2, 5, 8), unless the OMVs were first solubilized with the detergent Triton X-100 (Figure 3C, lanes 3, 6, 9) [51,52]. Therefore, display of heterologous β-helical passenger structures fused to HbpD(Δd1) was achieved at *E. coli* OMV surface, in contrast to what was found for PmpD (FL).

### 3.3. Display of Truncated Passengers upon Fusion to Hbp in Salmonella

**Figure 4:**
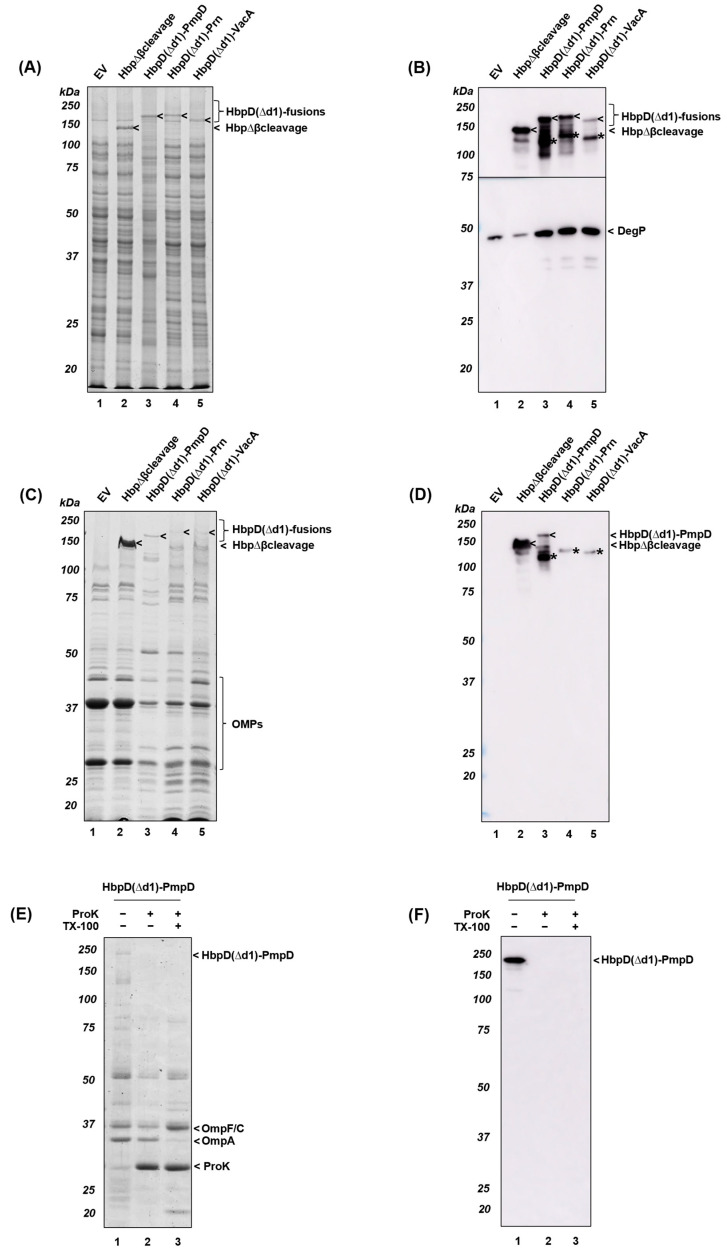
Expression of EV, HbpΔβcleavage, HbpD(Δd1)-PmpD, HbpD(Δd1)-Prn and HbpD(Δd1)-VacA in *Salmonella* and their OMVs. SL3261 Δ*tolRA* Δ*msbB* cells were induced for expression of empty vector pEH3 (EV), HbpΔβcleavage, HbpD(Δd1)-PmpD, HbpD(Δd1)-Prn and HbpD(Δd1)-VacA and whole cells were analyzed by SDS-PAGE followed by Coomassie staining (**A**) and immunoblotting using antisera against Hbp or DegP (**B**). Equivalent amounts of OMVs were analyzed by SDS-PAGE followed by Coomassie staining (**C**) or immunoblotting using Hbp antiserum (**D**). In the panel (**B**–**D**), bands representing proteolytically degraded HbpD(Δd1) were indicated (*). OMVs expressing HbpD(Δd1)-PmpD were used for a proteinase K (ProK) assay. Samples of untreated intact OMVs, intact OMVs treated with ProK, and lysed OMVs, treated with TritonX-100 (TX-100) and incubated with ProK were analyzed by SDS-PAGE followed by Coomassie staining (**E**) or immunoblotting using PmpD-directed antiserum (**F**). MW markers (in kDa) are indicated at the left and identified protein bands at the right side of panels.

Our laboratory prefers to use OMVs from the attenuated and hypervesiculating *Salmonella* Typhimurium strain SL3261 Δ*tolRA* Δ*msbB* as vaccine carrier, since it carries a detoxified version of LPS and was also previously used to display various HbpD(Δd1)-fusion constructs at the surface of OMVs [21,26]. We, therefore, tested expression of the HbpD(Δd1)-PmpD, -Prn and -VacA fusions in this strain and used as controls empty vector (pEH3-EV) and expression of HbpΔβcleavage [23], which displays the full-length Hbp passenger including domain 1. Following our routine production of OMVs [21,26], cells were induced overnight for protein expression with 0.1 mM IPTG, after which cells and OMVs were collected and analyzed (Figure 4). Expression of HbpD(Δd1)-PmpD, -Prn and to a lesser extent -VacA fusions in cells was observed on Coomassie-stained gels, albeit at lower levels than the HbpΔβcleavage positive control (Figure 4A). Immunoblotting using Hbp antiserum did confirm the expression of the HbpD(Δd1)-fusion products (Figure 4B), but also revealed lower running bands (*) that were likely degradation products of the HbpD(Δd1) fusions. Of note, the Coomassie-stained gel hardly displayed these undesirable HbpD(Δd1)-fusion truncates and it remains unclear why they are so prominently detected by immunoblotting (c.f. Figure 4A,B, lanes 3–5).

When analyzing the OMVs using Coomassie-stained gels and immunoblotting, HbpD(Δd1)-PmpD was clearly detected, although less than HbpΔβcleavage (Figure 4C,D, lanes 2–3). In contrast, HbpD(Δd1)-Prn and -VacA were not detected (Figure 4C,D, lanes 4–5). Furthermore, the translocation of HbpD(Δd1)-PmpD into OMVs appeared inefficient when compared to HbpΔβcleavage, especially when compared to their respective expression levels in cells (Figure 4A). This suggests that the impaired recruitment of the HbpD(Δd1)-fusions to OMVs is due to the presence of the heterologous passengers. To monitor surface display of HbpD(Δd1)-PmpD, the OMVs were also subjected to ProK treatment. HbpD(Δd1)-PmpD was readily degraded consistent with presentation at the OMV surface (Figure 4E, lane 2; Figure 4F, lane 2). As a control for *Salmonella* OMV integrity, we analyzed the ProK sensitivity of OmpA, which carries a protease-susceptible periplasmic domain. Indeed, OmpA was only degraded upon Triton X-100 (Figure 4A, c.f. lanes 2 and 3), confirming the integrity of OMVs [53].

Although the analyzed amounts of OMV material loaded on the gels were derived from the same amounts of *Salmonella* cells, as judged from OD_600_ densities (Figure 4C), the OMP protein profiles detected were considerably weaker in HbpD(Δd1)-PmpD, -Prn and -VacA OMVs when compared to HbpΔβcleavage and EV control OMVs (c.f Figure 4C, lanes 1–2 and lanes 3–5). This could reflect the downregulation of OMP protein expression, but considering the smaller pellets obtained upon ultracentrifugation (data not shown), the impaired vesiculation by cells expressing the heterologous HbpD(Δd1)-fusion constructs appears a more likely explanation. Possibly, this observation is connected to the induction of cellular stress indicated by elevated levels of the periplasmic protease DegP (Figure 4B, cf. lanes 1–2 and 3–5) [54]. In conclusion, display of truncated PmpD on *Salmonella* OMVs has been achieved but comes at the cost of decreased efficiency of OMV formation.

### 3.4. Display of Antigenic Passengers upon Seamless Fusion to the β-Helical Stem of Hbp

**Figure 5:**
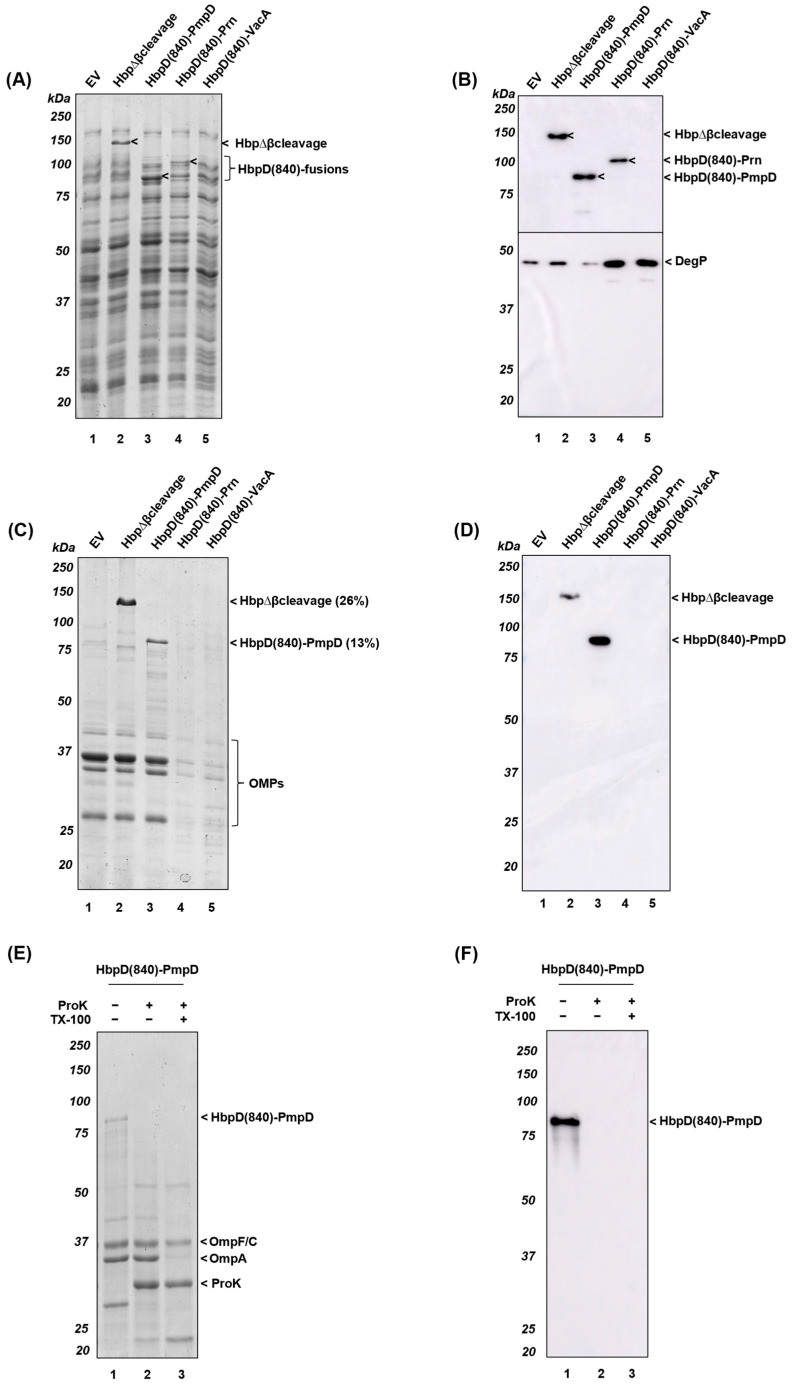
Expression of EV, HbpΔβcleavage, HbpD(840)-PmpD, HbpD(840)-Prn and HbpD(840)-VacA in *Salmonella* whole cell lysates and isolated *Salmonella* OMVs. SL3261 Δ*tolRA* Δ*msbB* cells were induced for expression in presence of the empty vector (EV) or the pEH3 plasmids encoding HbpΔβcleavage, HbpD(840)-PmpD, HbpD(840)-Prn and HbpD(840)-VacA. Whole cell lysates were analyzed by SDS-PAGE followed by Coomassie staining (**A**) or immunoblotting using antiserum against Hbp (**B**). Equivalent amounts of OMVs were analyzed by SDS-PAGE followed by Coomassie staining (**C**) or immunoblotting using antiserum against Hbp (**D**). OMVs expressing HbpD(840)-PmpD were used for a proteinase K (ProK) assay. Samples of untreated intact OMVs, intact OMVs treated with ProK and lysed OMVs, treated with TritonX-100 (TX-100) and incubated with ProK were analyzed by SDS-PAGE followed by Coomassie staining (**E**) or immunoblotting using Hbp antiserum (**F**). MW markers (in kDa) are indicated at the left and identified protein bands at the right side of panels.

Progressive stacking of parallel β-strands into a β-helical structure is thought to contribute to the driving force for translocation of AT passengers across the OM [55]. One explanation for the observed suboptimal display of heterologous passengers upon fusion to HbpD(Δd1), may be that β-helix formation is either interrupted or completely disturbed at the fusion point, which is a linker predicted to be unstructured (Figure S2B). To address this potential incompatibility with efficient folding during secretion, we used an alternative fusion approach that aimed to connect the β-helical PmpD, Prn and VacA passenger segments directly to the β-helical core of Hbp. Alphafold2 was used to test various in silico fusions to identify transition points that are modelled to yield a seamless uninterrupted chimeric β-helix [44]. This resulted in three fusion constructs in which the secreted and antigenic passenger fragments of PmpD (aa68-629), Prn (aa35-350) and VacA (aa65-377) were combined with a display version of HbpD from residue 840 onward (indicated as HbpD(840), see Figure S5A). These seamless fusions were expressed in *Salmonella* SL3261 Δ*tolRA* Δ*msbB* cells. This yielded bands of ~90 kDa for HbpD(840)-PmpD and ~100 kDa for HbpD(840)-Prn bands on Coomassie-stained gels that were also detected by immunoblotting using Hbp antiserum (Figure 5A,B, lanes 3–4). However, expression of the ~100 kDa HbpD(840)-VacA construct was not detected (Figure 5A,B, lane 5). Of note, HbpD(840)-PmpD expression in cells appeared more pronounced than the HbpΔβcleavage control analyzed in parallel (Figure 5A, lane 2) and, in line with an efficient biogenesis, no upregulation of the periplasmic stress factor DegP was observed (Figure 5B, lane 3). On the other hand, HbpD(840)-Prn and -VacA expression did result in elevated levels of DegP, indicative of cellular stress due to impaired translocation of the fusions across the CEs (Figure 5B, lanes 4–5).

OMVs were isolated from the various cultures and analyzed for the presence of the HbpD(840)-fusion constructs (Figure 5C,D). As observed for the HbpD(Δd1)-fusions, OMVs derived from cultures expressing the HbpD(840)-Prn and -VacA fusion constructs also showed lower levels of OMPs (Figure 5C, lanes 4–5), indicating lower OMV yields, again perhaps related to the CE stress induced (cf. Figure 4 and Figure 5). In contrast, OMV formation upon expression of the HbpD(840)-PmpD fusion appeared similar to the controls (Figure 5C, cf. lane 3 and lanes 1–2). Importantly, expression of HbpD(840)-PmpD in OMVs was highly efficient and was quantified to constitute ~13% of the total OMV protein content (Figure 5C,D, lane 3). In comparison, the HbpΔβcleavage control reached a level of ~26%, indicating that the presence of the PmpD segment still slightly hampered partitioning of the HbpD(840)-PmpD fusion into OMVs. Yet, considerable improvement over the HbpD(Δd1)-PmpD was achieved when regarding OMV yields and expression (Figure 4C, lane 3). Finally, display of HbpD(840)-PmpD at the OMV surface was confirmed by its ProK accessibility (Figure 5E,F, lane 5). Taken together, fusion to HbpD(840) allowed for efficient expression of the truncated PmpD antigen at the OMV surface. However, our results also indicate that we could not apply this approach as a generic fusion for other passenger fragments, such as Prn and VacA.

## 4. Discussion

In this work, we applied the HbpD display platform to successfully present a fragment of the *C. trachomatis* antigen PmpD at the surface of LPS-detoxified *Salmonella* OMVs. These recombinant OMVs are ready for use as an experimental mucosal vaccine in a mouse model. If effective, the OMV-based vaccine could be combined with other purified antigens such as MOMP to constitute a multivalent vaccine that benefits from the adjuvant properties of OMVs, a formulation strategy that is required for the effectivity of the 4CMenB vaccine (Bexsero, GSK) that protects against infections by *N. meningitidis* [20]. Importantly, the path towards construction of the recombinant OMVs provided insight in the experimental intricacies of expression of heterologous AT-derived antigens in bacterial OMVs.

Compared to other Gram-negative secretion systems, autotransport is relatively simple, featuring sequential transfer across the IM and OM involving the conserved Sec and Bam complexes, respectively [56,57]. Therefore, secretion or surface display of a specific AT could be feasible in any Gram-negative host, although species-specific targeting signals for the Sec and Bam machinery may influence the efficiency of the secretion process. Indeed, we achieved efficient expression and OM assembly in *E. coli* of *C. trachomatis* AT PmpD (FL), in which the C-terminal β-signal was optimized for recognition by the *E. coli* Bam complex. Expression did not increase in the BL21(DE3)omp8 strain, which lacks multiple high-level OMPs, indicating that the availability of the *E. coli* Bam complex is not a bottleneck for PmpD (FL) localization, or some other heterologous OMPs [40,58,59]. Surprisingly, however, PmpD (FL) was not included in OMVs pinching off from the BL21(DE3)omp8 surface (Figure 1E). This strain is hypervesiculating due to the absence of OmpA that normally provides anchoring of the OM to the periplasmic peptidoglycan layer. While it is known that the partitioning of OMPs into OMVs is not an entirely random process, the total exclusion observed is most likely related to structural features in PmpD (FL) that preclude its recruitment in OMVs. Possibly, a difference in thickness of the hydrophobic core of the *C. trachomatis* and *E.coli* OM bi-layers may constitute a mismatch between the relatively extended β-barrel structure in PmpD (FL) [60] and the thinner hydrophobic core of the *E. coli* OM, while being required for assembly into its native OM. Although little is known about the mechanism of formation of bacterial OMVs, this putative mismatch may cause a local inspissation of the *E. coli* OM that could interfere with OMV development.

To address this issue, we changed our strategy and fused a large immunogenic fragment of the extracellular passenger of PmpD via a linker to the *E. coli* AT Hbp, which has been applied before to display antigens on OMVs. The passenger of Hbp is anchored to the surface of OMVs by its cognate β-barrel domain as a result of an inactivated autoproteolytic cleavage site [22]. Despite the relatively large size of the PmpD fragment, we considered this approach promising in view of the conserved β-helical passenger structures shared between PmpD and Hbp (Figure S2B). Furthermore, we also fused passenger fragments of *B. pertussis* Prn and *H. pylori* VacA to examine the general applicability of this approach. The data showed that the truncated PmpD was included and exposed at the surface of BL21(DE3)omp8 OMVs (Figure 3), consistent with the notion that the *E. coli* Hbp β-barrel anchor being better suited for recruitment to OMVs than the β-barrel of *C. trachomatis* PmpD. Similar results were obtained for the HbpD(Δd1)-Prn construct, but OMVs carried only low amounts of HbpD(Δd1)-VacA probably due to poor expression levels (Figure S4B). Overall, the results suggest that subtle differences in the selected AT fragments or the connection to the HbpD(Δd1)-based platform may strongly influence expression and assembly in the *E. coli* OM and OMVs. This unexplained complexity is in line with our previous studies using ATs for surface display of heterologous antigens [23,61]. Often, inefficient expression of fusion constructs was observed, concomitant with toxicity and translocation incompetence.

We next expressed the HbpD(Δd1)-fusion constructs in a relevant hypervesiculating and LPS-detoxified *Salmonella* Typhimurium strain that is used to make vaccine-grade OMVs [21], which resulted in adequate expression levels of the HbpD(Δd1)-fusions. However, inclusion of HbpD(Δd1)-PmpD in *Salmonella* OMVs was relatively inefficient, and even undetectable for HbpD(Δd1)-Prn and -VacA (Figure 4C,D). Furthermore, unlike what was observed for BL21(DE3)omp8, expression of these HbpD(Δd1)-fusions in the *Salmonella* strain lowered the amounts of OMVs produced. This was unexpected, in view of the increased levels of periplasmic protease DegP, which indicates cell envelope stress, which normally induces OMV formation [62]. At present, we have no explanation for these, apparently strain-dependent, effects. In general, the *E. coli* and *Salmonella* strains used have a very similar genetic background, although it should be noted that the molecular basis of hypervesiculation of the strains differs. While hypervesiculation in BL21(DE3)omp8 is probably due to the absence of OmpA, the *Salmonella* strain lacks TolRA thereby disrupting a major link between the IM and OM [22,28].

In any case, expression of the HbpD(Δd1)-fusion constructs in the *Salmonella* vaccine strain was relatively poor. Possibly, the discontinuation of the β-helical stem structure by inclusion of the flexible linker between HbpD(Δ1) and its N-terminally fused heterologous partner disrupts sequential stacking of β-strands at the cell surface. This mechanism is thought to be at least partially responsible for energizing the translocation process [53,60] and point mutations in the C-terminal part of the β-helical stem of Hbp, which disrupt the initiation of stacking, have been shown to negatively influence secretion [63]. This may result in the formation of translocation intermediates that elicit cell envelope stress, consistent with the upregulated levels of the protease DegP observed. In an attempt to provide a smoother structural transition between the fusion partner and the displaying constructs, Alphafold2 prediction was used to select fusions that are modelled to seamlessly stitch the β-strands of the heterologous passenger into the β-helical stem of the Hbp carrier (Figure S5A). This approach appeared successful for the HbpD(840)-PmpD fusion, as implied by the decreased levels of stress and degradation observed, and the detection of higher levels of PmpD at the surface of OMVs (Figure 5C,D). Furthermore, the efficiency of OMV formation appeared restored, providing a solid basis to evaluate the safety and immunogenicity of HbpD(840)-PmpD-containing OMVs as a mucosal vaccine in future studies. In marked contrast, the predicted seamless HbpD(840)-Prn and -VacA fusions still caused stress and resulted in lower production of OMVs that also did not contain detectable amounts of these fusions.

## 5. Conclusions

In conclusion, while tailoring the HbpD-fusion strategies may improve expression of individual AT antigens at the surface of OMVs, this has to be addressed on a case-to-case basis. In this study, six chimeric constructs were evaluated. Alternatively, a more random fusion approach combined with a screening for improved display could be considered, until a more rational approach is found, based on evidence as to why some fusions are successful and others not. As for non-AT antigens, SpyTag/SpyCatcher coupling is the safest bet for efficient display on OMVs, but this approach comes at the cost of a more complex, hence expensive manufacturing process.

## Data Availability

The data presented in this study are contained within the article and the available supplementary material.

## Supplementary Materials

The following supporting information can be downloaded at: https://www.mdpi.com/article/10.3390/membranes13040366/s1.
