# Supplementary material for: Heterologous Display of Chlamydia trachomatis PmpD Passenger at the Surface of Salmonella OMVs
Source: Membranes (Basel). 2023 Mar 23;13(4):366. doi: 10.3390/membranes13040366 (PMC10145130; doi:10.3390/membranes13040366)
Supplement: Supplementary file 1 [file membranes-13-00366-s001.zip › membranes-2274865-supplementary.pdf]

Tabel S1: Primers used in this study

No.	Primers	Sequence 5'-3’
1	*Sac*I-PmpD (aa68-698)-fw	GGAAGTCTTGCGGGGAGCTCCGACAGCCAAGCGGAGGG
2	*BamH*I-PmpD (aa68-698)-rv	TACCGCTGCCGGATCCACGAAGGGACGAGATAGCTCC
3	*Sac*I-Prn (aa35-574)-fw	GGAAGTCTTGCGGGGAGCTCCATCAAGGTAAGCGGCCGT
4	*BamH*I-Prn (aa35-574)-rv	TACCGCTGCCGGATCCGGACAACTCCCTGCCCGC
5	*Sac*I-VacA (aa65-493)-fw	GGAAGTCTTGCGGGGAGCTCCAAACAAGCCGAAGAAGCGAATAAAACC
6	*BamH*I-VacA (aa65-493)-rv	TACCGCTGCCGGATCCTCCCAAACTAATATCGTTATTGAAAGTGGC
7	*Sac*I-PmpD (aa68-629)-fw	CTGCAGGAAGTCTTGCGGGGAGCTCCGACAGCCAAGCGGAG
8	PmpD (aa68-629)-rv	GGGTGGCATCGGAGCTAATCGCGCCGCCGCCTAA
9	“PmpD”-HbpD (840)-fw	GATTAGCTCCGATGCCACCCTGAGTC
10	*Kpn*I-HbpD (921)-rv	CTCCCGCTCCAGATATTGCGGTACCCGTTAAACAGGCTGTA
11	*Sac*I-Prn (aa35-350)-fw	CTGCAGGAAGTCTTGCGGGGAGCTCCATCAAGGTAAGCGGCCGT
12	Prn (aa35-350)-rv	GTGGCATCGGCCAGCGCCACGTCGAG
13	“Prn”-HbpD (840)-fw	GTGGCGCTGGCCGATGCCACCCTGAGTCTGAAC
14	*Sac*I-VacA (aa65-377)-fw	CTGCAGGAAGTCTTGCGGGGAGCTCCAAACAAGCCGAAGAAGCG
15	VacA (aa65-377)-rv	GGGTGGCATCATTTTCCACTAAAAGGGTG
16	“VacA”-HbpD (840)-fw	AGTGGAAAATGATGCCACCCTGAGTCTGAAC

Fasta files of amino acid sequences used in this study as expressed in *E. coli*.

Colorcoding is used to indicate significant features, which is explained under the sequences

>Amino acid sequence of *C. trachomatis* serovar L2 PmpD (wild-type); (strain 434/Bu/ATCC)

MSSEKDIKSTCSKFSLSVVAAILASVSGLASCVDLHAGGQSVNELVYVGPQAVLLLDQIRDLFVGSKDS
QAEGQYRLIVGDPSSFQEKDADTLPGKVEQSTLFSVTNPVVFQGVDQQDQVSSQGLICSFTSSNLDSPR
DGESFLGVAFVGDSSKAGITLTDVKASLSGAALYSTEDLIFEKIKGGLEFASCSSLEQGGACAAQSILIHD
CQGLQVKHCTTAVNAEGSSANDHLGFGGGAFFVTGSLSGEKSLYMPAGDMVVANCDGAISFEGNSAN
FANGGAIAASGKVLFVANDKKTSFIENRALSGGAIAASSDIAFQNCAELVFKGNCAIGTEDKGSLGGGA
ISSLGTVLLQGNHGITCDKNESASQGGAIFGKNCQISDNEGPVVFRDSTACLGGGAIAAQEIVSIQNNQA
GISFEGGKASFGGGIACGSFSSAGGASVLGTIDISKNLGAISFSRTLCTTSDLGQMEYQGGGALFGENISL
SENAGVLTFKDNIVKTFASNGKILGGGAILATGKVEITNNSEGISFTGNARAPQALPTQEEFPLFSKKEG
RPLSSGYSGGGAILGREVAILHNAAVVFEQNRLQCSEEEATLLGCCGGGAVHGMDSTSIVGNSSVRFG
NNYAMGQGVSGGALLSKTVQLAGNGSVDFSRNIASLGGGALQASEGNCELVDNGYVLFRDNRGRVY
GGAISCLRGDVVISGNKGRVEFKDNIATRLYVEETVEKVEEVEPAPEQKDNNELSFLGSAEQSFITAAN
QALFASEDGDLSPESSISSEELAKRRECAGGAIFAKRVRIVDNQEAVVFSNNFSDIYGGAIFTGSLREEDK
LDGQIPEVLISGNAGDVVFSGNSSKRDEHLPHTGGGAICTQNLTISQNTGNVLFYNNVACSGGAVRIED
HGNVLLEAFGGDIVFKGNSSFRAQGSDAIYFAGKESHITALNATEGHAIVFHDALVFENLEERKSAEVL
LINSRENPGYTGSIRFLEAESKVPQCIHVQQGSLELLNGATLCSYGFKQDAGAKLVLAAGAKLKILDSG
TPVQQGHAISKPEAEIESSSEPEGAHSLWIAKNAQTTVPMVDIHTISVDLASFSSSQQEGTVEAPQVIVPG
GSYVRSGELNLELVNTTGTGYENHALLKNEAKVPLMSFVASGDEASAEISNLSVSDLQIHVVTPEIEED
TYGHMGDWSEAKIQDGTLVISWNPTGYRLDPQKAGALVFNALWEEGAVLSALKNARFAHNLTAQRM
EFDYSTNVWGFAFGGFRTLSAENLVAIDGYKGAYGGASAGVDIQLMEDFVLGVSGAAFLGKMDSQKF
DAEVSRKGVVGSVYTGFLAGSWFFKGQYSLGETQNDMKTRYGVLGESSASWTSRGVLADALVEYRS
LVGPVRPTFYALHFNPYVEVSYASMKFPGFTEQGREARSFEDASLTNITIPLGMKFELAFIKGQFSEVNS
LGISYAWEAYRKVEGGAVQLLEAGFDWEGAPMDLPRQELRVALENNTEWSSYFSTVLGLTAFCGGFT
STDSKLGYEANTGLRLIF

>Amino acid sequence of PmpD (full-length)

MNRIYSLRYSAVARGFIAVSEFARKCVHKSVRRLCFPVLLLIPVLFSAGSLAGTVNNELGSGSGYPYDV
PDYAGSGTSSSVDLHAGGQSVNELVYVGPQAVLLLDQIRDLFVGSKDSQAEGQYRLIVGDPSSFQEKD
ADTLPGKVEQSTLFSVTNPVVFQGVDQQDQVSSQGLISSFTSSNLDSPRDGESFLGIAFVGDSSKAGITL
TDVKASLSGAALYSTEDLIFEKIKGGLEFASSSSLEQGGASAAQSILIHDSQGLQVKHSTTAVNAEGSSA
NDHLGFGGGAFFVTGSLSGEKSLYMPAGDMVVANSDGAISFEGNSANFANGGAIAASGKVLFVANDK
KTSFIENRALSGGAIAASSDIAFQNSAELVFKGNSAIGTEDKGSLGGGAISSLGTVLLQGNHGITSDKNES
ASQGGAIFGKNSQISDNEGPVVFRDSTASLGGGAIAAQEIVSIQNNQAGISFEGGKASFGGGIASGSFSSA
GGASVLGTIDISKNLGAISFSRTLSTTSDLGQMEYQGGGALFGENISLSENAGVLTFKDNIVKTFASNGKI
LGGGAILATGKVEITNNSGGISFTGNARAPQALPTQEEFPLFSKKEGRPLSSGYSGGGAILGREVAILHN
AAVVFEQNRLQSSEEEATLLGSSGGGAVHGMDSTSIVGNSSVRFGNNYAMGQGVSGGALLSKTVQLA
GNGSVDFSRNIASLGGGALQASEGNSELVDNGYVLFRDNRGRVYGGAISSLRGDVVISGNKGRVEFKD
NIATRLYVEETVEKVEEVEPAPEQKDNNELSFLGSVEQSFITAANQALFASEDGDLSPESSISSEELAKRR
ESAGGAIFAKRVRIVDNQEAVVFSNNFSDIYGGAIFTGSLREEDKLDGQIPEVLISGNAGDVVFSGNSSK
RDEHLPHTGGGAISTQNLTISQNTGNVLFYNNVASSGGAVRIEDHGNVLLEAFGGDIVFKGNSSFRAQG
SDAIYFAGKESHITALNATEGHAIVFHDALVFENLKERKSAEVLLINSRENPGYTGSIRFLEAESKVPQSI
HVQQGSLELLNGATLSSYGFKQDAGAKLVLAAGSKLKILDSGTPVQGHAISKPEAEIESSSEPEGAHSL
WIAKNAQTTVPMVDIHTISVDLASFSSSQQEGTVEAPQVIVPGGSYVRSGELNLELVNTTGTGYENHAL
LKNEAKVPLMSFVASSDEASAEISNLSVSDLQIHVATPEIEEDTYGHMGDWSEAKIQDGTLVINWNPTG
YRLDPQKAGALVFNALWEEGAVLSALKNARFAHNLTAQRMEFDYSTNVWGFAFGGFRTLSAENLVAI
DGYKGAYGGASAGVDIQLMEDFVLGVSGAAFLGKMDSQKFDAEVSRKGVVGSVYTGFLAGSWFFKG
QYSLGETQNDMKTRYGVLGESSASWTSRGVLADALVEYRSLVGPVRPTFYALHFNPYVEVSYASMKF
PGFTEQGREARSFEDASLTNITIPLGMKFELAFIKGQFSEVNSLGISYAWEAYRKVEGGAVQLLEAGFD
WEGAPMDLPRQELRVALENNTEWSSYFSTVLGLTAFSGGFTSTDSKLGYEANAGLRYSF

E. coli Hbp signal peptide

Hbp N-terminal (passenger domain) ; GTVNN: Edman N-terminal amino acid sequencing result

HA tag;

C. trachomatis serovar L2 PmpD (full-length) with 24 cysteine (C) residues replaced by Serine (S): underlined

Hbp (RYSF) conserved C-terminal motif

>Amino acid sequence of HbpD(Δd1)-PmpD (aa68-698)

MNRIYSLRYSAVARGFIAVSEFARKCVHKSVRRLCFPVLLLIPVLFSAGSLAGSSDSQAEGQYRLIVGDP
SSFQEKDADTLPGKVEQSTLFSVTNPVVFQGVDQQDQVSSQGLISSFTSSNLDSPRDGESFLGIAFVGDS
SKAGITLTDVKASLSGAALYSTEDLIFEKIKGGLEFASSSSLEQGGASAAQSILIHDSQGLQVKHSTTAVN
AEGSSANDHLGFGGGAFFVTGSLSGEKSLYMPAGDMVVANSDGAISFEGNSANFANGGAIAASGKVLF
VANDKKTSFIENRALSGGAIAASSDIAFQNSAELVFKGNSAIGTEDKGSLGGGAISSLGTVLLQGNHGIT
SDKNESASQGGAIFGKNSQISDNEGPVVFRDSTASLGGGAIAAQEIVSIQNNQAGISFEGGKASFGGGIA
SGSFSSAGGASVLGTIDISKNLGAISFSRTLSTTSDLGQMEYQGGGALFGENISLSENAGVLTFKDNIVKT
FASNGKILGGGAILATGKVEITNNSGGISFTGNARAPQALPTQEEFPLFSKKEGRPLSSGYSGGGAILGRE
VAILHNAAVVFEQNRLQSSEEEATLLGSSGGGAVHGMDSTSIVGNSSVRFGNNYAMGQGVSGGALLS
KTVQLAGNGSVDFSRNIASLGGGALQASEGNSELVDNGYVLFRDNRGRVYGGAISSLRGSGSGNDAPV
TFRTSEGGALEWSFNSSTGAGALTQGTTTYAMHGQQGNDLNAGKNLIFQGQNGQINLKDSVSQGAGS
LTFRDNYTVTTSNGSTWTGAGIVVDNGVSVNWQVNGVKGDNLHKIGEGTLTVQGTGINEGGLKVGD
GKVVLNQQADNKGQVQAFSSVNIASGRPTVVLTDERQVNPDTVSWGYRGGTLDVNGNSLTFHQLKA

ADYGAVLANNVDKRATITLDYALRADKVALNGWSESGKGTAGNLYKYNNPYTNTTDYFILKQSTYG
YFPTDQSSNATWEFVGHSQGDAQKLVADRFNTAGYLFHGQLKGNLNVDNRLPEGVTGALVMDGAAD
ISGTFTQENGRLTLQGHPVIHAYNTQSVADKLAASGDHSVLTQPTSFSQEDWENRSFTFDRLSLKNTDF
GLGRNATLNTTIQADNSSVTLGDSRVFIDKNDGQGTAFTLEEGTSVATKDADKSVFNGTVNLDNQSVL
NINDIFNGGIQANNSTVNISSDSAVLGNSTLTSTALNLNKGANALASQSFVSDGPVNISDATLSLNSRPD
EVSHTLLPVYDYAGSWNLKGDDARLNVGPYSMLSGNINVQDKGTVTLGGEGELSPDLTLQNQMLYSL
FNGYRNIWSGSLNAPDATVSMTDTQWSMNGNSTAGNMKLNRTIVGFNGGTSPFTTLTTDNLDAVQSA
FVMRTDLNKADKLVINKSATGHDNSIWVNFLKKPSNKDTLDIPLVSAPEATADNLFRASTRVVGFSDV
TPILSVRKEDGKKEWVLDGYQVARNDGQGKAAATFMHISYNNFITEVGSLNKRMGDLRDINGEAGTW
VRLLNGSGSADGGFTDHYTLLQMGADRKHELGSMDLFTGVMATYTDTDASADLYSGKTKSWGGGFY
ASGLFRSGAYFDVIAKYIHNENKYDLNFAGAGKQNFRSHSLYAGAEVGYRYHLTDTTFVEPQAELVW
GRLQGQTFNWNDSGMDVSMRRNSVNPLVGRTGVVSGKTFSGKDWSLTARAGLHYEFDLTDSADVHL
KDAAGEHQINGRKDSRMLYGVGLNARFGDNTRLGLEVERSAFGKYNTDDAINANIRYSF

E. coli Hbp signal peptide
C. trachomatis PmpD (aa68-698)
Flexible linker
E. coli HbpD

>Amino acid sequence of HbpD(Δd1)-Prn (aa35-574)
MNRIYSLRYSAVARGFIAVSEFARKCVHKSVRRLCFPVLLLIPVLFSAGSLAGSSIKVSGRQAQGILLENP
AAELQFRNGSVTSSGQLSDDGIRRFLGTVTVKAGKLVADHATLANVGDTWDDDGIALYVAGEQAQAS
IADSTLQGAGGVQIERGANVTVQRSAIVDGGLHIGALQSLQPEDLPPSRVVLRDTNVTAVPASGAPAAV
SVLGASELTLDGGHITGGRAAGVAAMQGAVVHLQRATIRRGDAPAGGAVPGGAVPGGAVPGGFGPG
GFGPVLDGWYGVDVSGSSVELAQSIVEAPELGAAIRVGRGARVTVSGGSLSAPHGNVIETGGARRFAP
QAAPLSITLQAGAHAQGKALLYRVLPEPVKLTLTGGADAQGDIVATELPSIPGTSIGPLDVALASQARW
TGATRAVDSLSIDNATWVMTDNSNVGALRLASDGSVDFQQPAEAGRFKVLTVNTLAGSGLFRMNVFA
DLGLSDKLVVMQDASGQHRLWVRNSGSEPASANTLLLVQTPLGSAATFTLANKDGKVDIGTYRYRLA
ANGNGQWSLVGAKAPPAPKPAPQPGPQPPQPPQPQPEAPAPQPPAGRELSGSGSGNDAPVTFRTSEGG
ALEWSFNSSTGAGALTQGTTTYAMHGQQGNDLNAGKNLIFQGQNGQINLKDSVSQGAGSLTFRDNYT
VTTSNGSTWTGAGIVVDNGVSVNWQVNGVKGDNLHKIGEGTLTVQGTGINEGGLKVGDGKVVLNQQ
ADNKGQVQAFSSVNIASGRPTVVLTDERQVNPDTVSWGYRGGTLDVNGNSLTFHQLKAADYGAVLA
NNVDKRATITLDYALRADKVALNGWSESGKGTAGNLYKYNNPYTNTTDYFILKQSTYGYFPTDQSSN
ATWEFVGHSQGDAQKLVADRFNTAGYLFHGQLKGNLNVDNRLPEGVTGALVMDGAADISGTFTQEN
GRLTLQGHPVIHAYNTQSVADKLAASGDHSVLTQPTSFSQEDWENRSFTFDRLSLKNTDFGLGRNATL
NTTIQADNSSVTLGDSRVFIDKNDGQGTAFTLEEGTSVATKDADKSVFNGTVNLDNQSVLNINDIFNGG
IQANNSTVNISSDSAVLGNSTLTSTALNLNKGANALASQSFVSDGPVNISDATLSLNSRPDEVSHTLLPV
YDYAGSWNLKGDDARLNVGPYSMLSGNINVQDKGTVTLGGEGELSPDLTLQNQMLYSLFNGYRNIW
SGSLNAPDATVSMTDTQWSMNGNSTAGNMKLNRTIVGFNGGTSPFTTLTTDNLDAVQSAFVMRTDLN
KADKLVINKSATGHDNSIWVNFLKKPSNKDTLDIPLVSAPEATADNLFRASTRVVGFSDVTPILSVRKE
DGKKEWVLDGYQVARNDGQGKAAATFMHISYNNFITEVGSLNKRMGDLRDINGEAGTWVRLLNGSG
SADGGFTDHYTLLQMGADRKHELGSMDLFTGVMATYTDTDASADLYSGKTKSWGGGFYASGLFRSG
AYFDVIAKYIHNENKYDLNFAGAGKQNFRSHSLYAGAEVGYRYHLTDTTFVEPQAELVWGRLQGQTF
NWNDSGMDVSMRRNSVNPLVGRTGVVSGKTFSGKDWSLTARAGLHYEFDLTDSADVHLKDAAGEH
QINGRKDSRMLYGVGLNARFGDNTRLGLEVERSAFGKYNTDDAINANIRYSF

E. coli Hbp signal peptide
B. pertussis Prn (aa35-574)
GSGSG: flexible linker
E. coli HbpD

>Amino acid sequence of HbpD(Δd1)-VacA (aa65-493)
MNRIYSLRYSAVARGFIAVSEFARKCVHKSVRRLCFPVLLLIPVLFSAGSLAGSSKQAEEANKTPDKPD
KVWRIQAGKGFNEFPNKEYDLYKSLLSSKIDGGWDWGNAARHYWVKGGQWNKLEVDMKDAVGTY
KLSGLRNFTGGDLDVNMQKATLRLGQFNGNSFTSYKDSADRTTRVNFNAKNISIDNFVEINNRVGSGA
GRKASSTVLTLQASEGITSSKNAEISLYDGATLNLASNSVKLNGNVWMGRLQYVGAYLAPSYSTINTS
KVQGEVDFNHLTVGDQNAAQAGIIASNKTHIGTLDLWQSAGLNIIAPPEGGYKDKPNSTTSQSGTKND
KKEISQNNNSNTEVINPPNNTQKTETEPTQVIDGPFAGGKDTVVNIFHLNTKADGTIKVGGFKASLTTN
AAHLNIGKGGVNLSNQASGRTLLVENLTGNITVDGPLRVNNQVGGYALAGSSANFEFKAGVDTKNGT
ATFNNDISLGGSGSGNDAPVTFRTSEGGALEWSFNSSTGAGALTQGTTTYAMHGQQGNDLNAGKNLIF
QGQNGQINLKDSVSQGAGSLTFRDNYTVTTSNGSTWTGAGIVVDNGVSVNWQVNGVKGDNLHKIGE
GTLTVQGTGINEGGLKVGDGKVVLNQQADNKGQVQAFSSVNIASGRPTVVLTDERQVNPDTVSWGY
RGGTLDVNGNSLTFHQLKAADYGAVLANNVDKRATITLDYALRADKVALNGWSESGKGTAGNLYKY
NNPYTNTTDYFILKQSTYGYFPTDQSSNATWEFVGHSQGDAQKLVADRFNTAGYLFHGQLKGNLNVD
NRLPEGVTGALVMDGAADISGTFTQENGRLTLQGHPVIHAYNTQSVADKLAASGDHSVLTQPTSFSQE
DWENRSFTFDRLSLKNTDFGLGRNATLNTTIQADNSSVTLGDSRVFIDKNDGQGTAFTLEEGTSVATKD
ADKSVFNGTVNLDNQSVLNINDIFNGGIQANNSTVNISSDSAVLGNSTLTSTALNLNKGANALASQSFV
SDGPVNISDATLSLNSRPDEVSHTLLPVYDYAGSWNLKGDDARLNVGPYSMLSGNINVQDKGTVTLG
GEGELSPDLTLQNQMLYSLFNGYRNIWSGSLNAPDATVSMTDTQWSMNGNSTAGNMKLNRTIVGFNG
GTSPFTTLTTDNLDAVQSAFVMRTDLNKADKLVINKSATGHDNSIWVNFLKKPSNKDTLDIPLVSAPEA
TADNLFRASTRVVGFSDVTPILSVRKEDGKKEWVLDGYQVARNDGQGKAAATFMHISYNNFITEVGSL
NKRMGDLRDINGEAGTWVRLLNGSGSADGGFTDHYTLLQMGADRKHELGSMDLFTGVMATYTDTD
ASADLYSGKTKSWGGGFYASGLFRSGAYFDVIAKYIHNENKYDLNFAGAGKQNFRSHSLYAGAEVGY
RYHLTDTTFVEPQAELVWGRLQGQTFNWNDSGMDVSMRRNSVNPLVGRTGVVSGKTFSGKDWSLTA
RAGLHYEFDLTDSADVHLKDAAGEHQINGRKDSRMLYGVGLNARFGDNTRLGLEVERSAFGKYNTD
DAINANIRYSF

E. coli Hbp signal peptide
H. pylori VacA (aa65-493)
GSGSG: flexible linker
E. coli HbpD

>Amino acid sequence of HbpD(840)-PmpD (aa68-629)
MNRIYSLRYSAVARGFIAVSEFARKCVHKSVRRLCFPVLLLIPVLFSAGSLAGSSDSQAEGQYRLIVGDP
SSFQEKDADTLPGKVEQSTLFSVTNPVVFQGVDQQDQVSSQGLISSFTSSNLDSPRDGESFLGIAFVGDS
SKAGITLTDVKASLSGAALYSTEDLIFEKIKGGLEFASSSSLEQGGASAAQSILIHDSQGLQVKHSTTAVN
AEGSSANDHLGFGGGAFFVTGSLSGEKSLYMPAGDMVVANSDGAISFEGNSANFANGGAIAASGKVLF
VANDKKTSFIENRALSGGAIAASSDIAFQNSAELVFKGNSAIGTEDKGSLGGGAISSDATLSLNSRPDEV
SHTLLPVYDYAGSWNLKGDDARLNVGPYSMLSGNINVQDKGTVTLGGEGELSPDLTLQNQMLYSLFN
GYRNIWSGSLNAPDATVSMTDTQWSMNGNSTAGNMKLNRTIVGFNGGTSPFTTLTTDNLDAVQSAFV
MRTDLNKADKLVINKSATGHDNSIWVNFLKKPSNKDTLDIPLVSAPEATADNLFRASTRVVGFSDVTPI
LSVRKEDGKKEWVLDGYQVARNDGQGKAAATFMHISYNNFITEVGSLNKRMGDLRDINGEAGTWVR

LLNGSGSADGGFTDHYTLLQMGADRKHELGSMDLFTGVMATYTDTDASADLYSGKTKSWGGGFYAS
GLFRSGAYFDVIAKYIHNENKYDLNFAGAGKQNFRSHSLYAGAEVGYRYHLTDTTFVEPQAELVWGR
LQGQTFNWNDSGMDVSMRRNSVNPLVGRTGVVSGKTFSGKDWSLTARAGLHYEFDLTDSADVHLKD
AAGEHQINGRKDSRMLYGVGLNARFGDNTRLGLEVERSAFGKYNTDDAINANIRYSF

E. coli Hbp signal peptide
C. trachomatis PmpD (aa68-698)
E. coli HbpD(840)

>Amino acid sequence of HbpD(840)-Prn (aa35-350)
MNRIYSLRYSAVARGFIAVSEFARKCVHKSVRRLCFPVLLLIPVLFSAGSLAGSSIKVSGRQAQGILLENP
AAELQFRNGSVTSSGQLSDDGIRRFLGTVTVKAGKLVADHATLANVGDTWDDDGIALYVAGEQAQAS
IADSTLQGAGGVQIERGANVTVQRSAIVDGGLHIGALQSLQPEDLPPSRVVLRDTNVTAVPASGAPAAV
SVLGASELTLDGGHITGGRAAGVAAMQGAVVHLQRATIRRGDAPAGGAVPGGAVPGGAVPGGFGPG
GFGPVLDGWYGVDVSGSSVELAQSIVEAPELGAAIRVGRGARVTVSGGSLSAPHGNVIETGGARRFAP
QAAPLSITLQAGAHAQGKALLYRVLPEPVKLTLTGGADAQGDIVATELPSIPGTSIGPLDVALADATLSL
NSRPDEVSHTLLPVYDYAGSWNLKGDDARLNVGPYSMLSGNINVQDKGTVTLGGEGELSPDLTLQNQ
MLYSLFNGYRNIWSGSLNAPDATVSMTDTQWSMNGNSTAGNMKLNRTIVGFNGGTSPFTTLTTDNLD
AVQSAFVMRTDLNKADKLVINKSATGHDNSIWVNFLKKPSNKDTLDIPLVSAPEATADNLFRASTRVV
GFSDVTPILSVRKEDGKKEWVLDGYQVARNDGQGKAAATFMHISYNNFITEVGSLNKRMGDLRDING
EAGTWVRLLNGSGSADGGFTDHYTLLQMGADRKHELGSMDLFTGVMATYTDTDASADLYSGKTKS
WGGGFYASGLFRSGAYFDVIAKYIHNENKYDLNFAGAGKQNFRSHSLYAGAEVGYRYHLTDTTFVEP
QAELVWGRLQGQTFNWNDSGMDVSMRRNSVNPLVGRTGVVSGKTFSGKDWSLTARAGLHYEFDLT
DSADVHLKDAAGEHQINGRKDSRMLYGVGLNARFGDNTRLGLEVERSAFGKYNTDDAINANIRYSF

E. coli Hbp signal peptide
B. pertussis Prn (aa35-350)
E. coli HbpD(840)

>Amino acid sequence of HbpD(840)-VacA (aa65-377)
MNRIYSLRYSAVARGFIAVSEFARKCVHKSVRRLCFPVLLLIPVLFSAGSLAGSSKQAEEANKTPDKPD
KVWRIQAGKGFNEFPNKEYDLYKSLLSSKIDGGWDWGNAARHYWVKGGQWNKLEVDMKDAVGTY
KLSGLRNFTGGDLDVNMQKATLRLGQFNGNSFTSYKDSADRTTRVNFNAKNISIDNFVEINNRVGSGA
GRKASSTVLTLQASEGITSSKNAEISLYDGATLNLASNSVKLNGNVWMGRLQYVGAYLAPSYSTINTS
KVQGEVDFNHLTVGDQNAAQAGIIASNKTHIGTLDLWQSAGLNIIAPPEGGYKDKPNSTTSQSGTKND
KKEISQNNNSNTEVINPPNNTQKTETEPTQVIDGPFAGGKDTVVNIFHLNTKADGTIKVGGFKASLTTN
AAHLNIGKGGVNLSNQASGRTLLVENDATLSLNSRPDEVSHTLLPVYDYAGSWNLKGDDARLNVGPY
SMLSGNINVQDKGTVTLGGEGELSPDLTLQNQMLYSLFNGYRNIWSGSLNAPDATVSMTDTQWSMNG
NSTAGNMKLNRTIVGFNGGTSPFTTLTTDNLDAVQSAFVMRTDLNKADKLVINKSATGHDNSIWVNFL
KKPSNKDTLDIPLVSAPEATADNLFRASTRVVGFSDVTPILSVRKEDGKKEWVLDGYQVARNDGQGK
AAATFMHISYNNFITEVGSLNKRMGDLRDINGEAGTWVRLLNGSGSADGGFTDHYTLLQMGADRKHE
LGSMDLFTGVMATYTDTDASADLYSGKTKSWGGGFYASGLFRSGAYFDVIAKYIHNENKYDLNFAGA
GKQNFRSHSLYAGAEVGYRYHLTDTTFVEPQAELVWGRLQGQTFNWNDSGMDVSMRRNSVNPLVGR
TGVVSGKTFSGKDWSLTARAGLHYEFDLTDSADVHLKDAAGEHQINGRKDSRMLYGVGLNARFGDN
TRLGLEVERSAFGKYNTDDAINANIRYSF

E. coli Hbp signal peptide

H. pylori VacA (aa65-377)

E. coli HbpD(840)

Supplimentary figures

Fig. S1

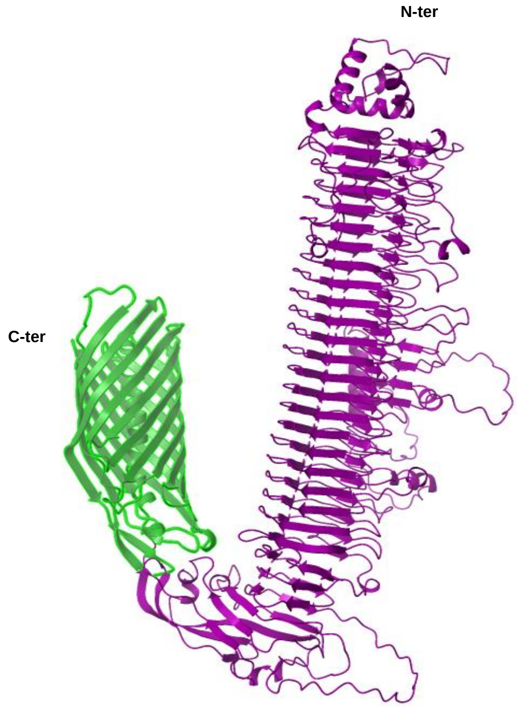

Fig. S1: Structural model of *C. trachomatis* PmpD (FL), serovar L2 (strain 434/Bu/ATCC VR-902B). Cartoon representation for the N-terminal β-helical passenger (purple) and C-terminal β-barrel of PmpD (FL), predicted by Alphafold2.

Fig. S2

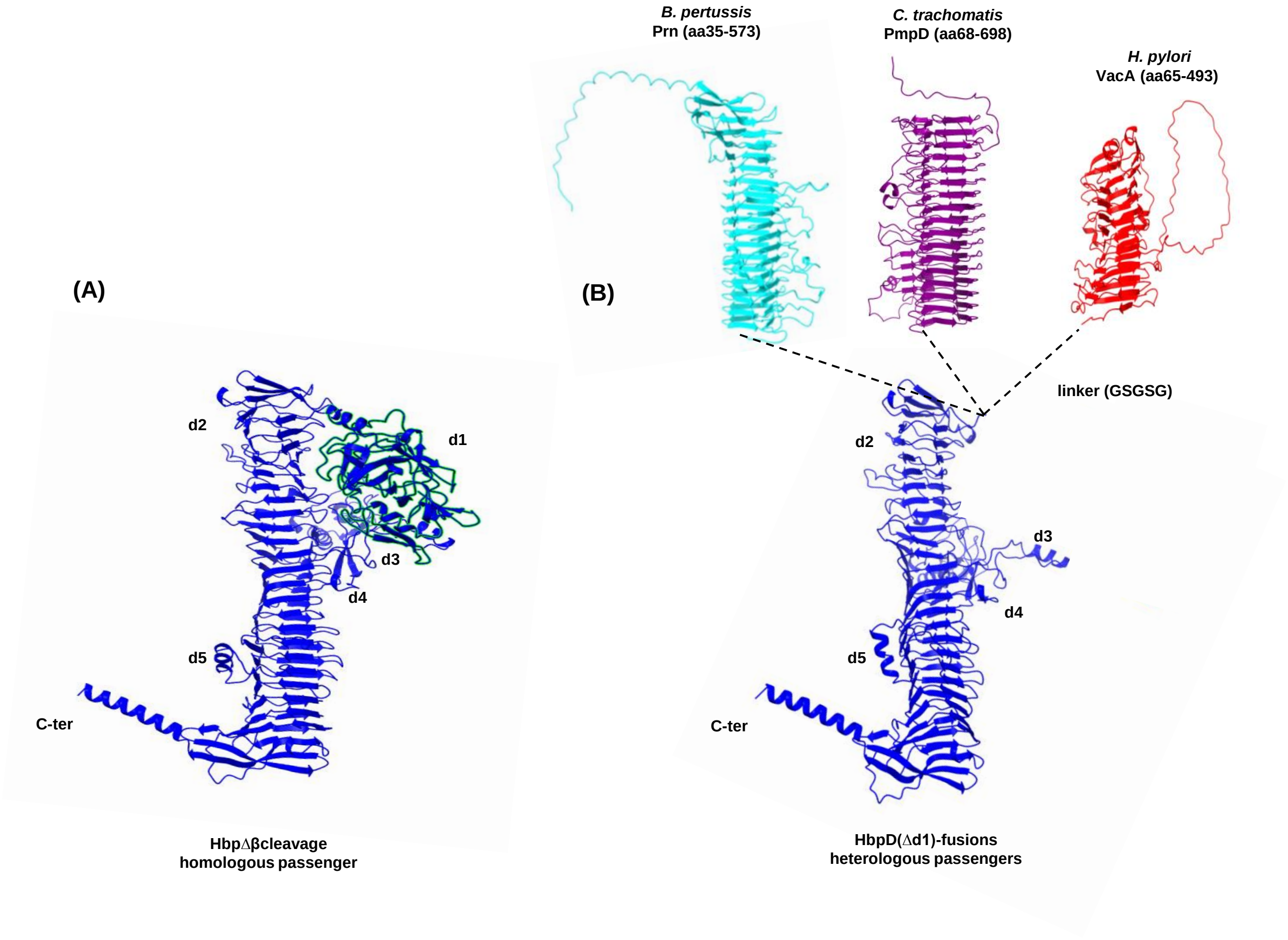

Fig. S2: Structural models of HbpΔβcleavage and HbpD(Δd1)-fusion passengers. Cartoon representation of homologous passenger HbpΔβcleavage, containing five side-domains d1-d5 (**A**) and of heterologous passengers HbpD(Δd1)-PmpD (aa68-698), HbpD(Δd1)-Prn (aa35-573) and HbpD(Δd1)-VacA (aa65-493) (**B**) predicted by Alphafold2. In the panel B, the flexible fusion linker (GSGSG) between HbpD(Δd1) and the fused truncated passengers were indicated.

Fig. S3

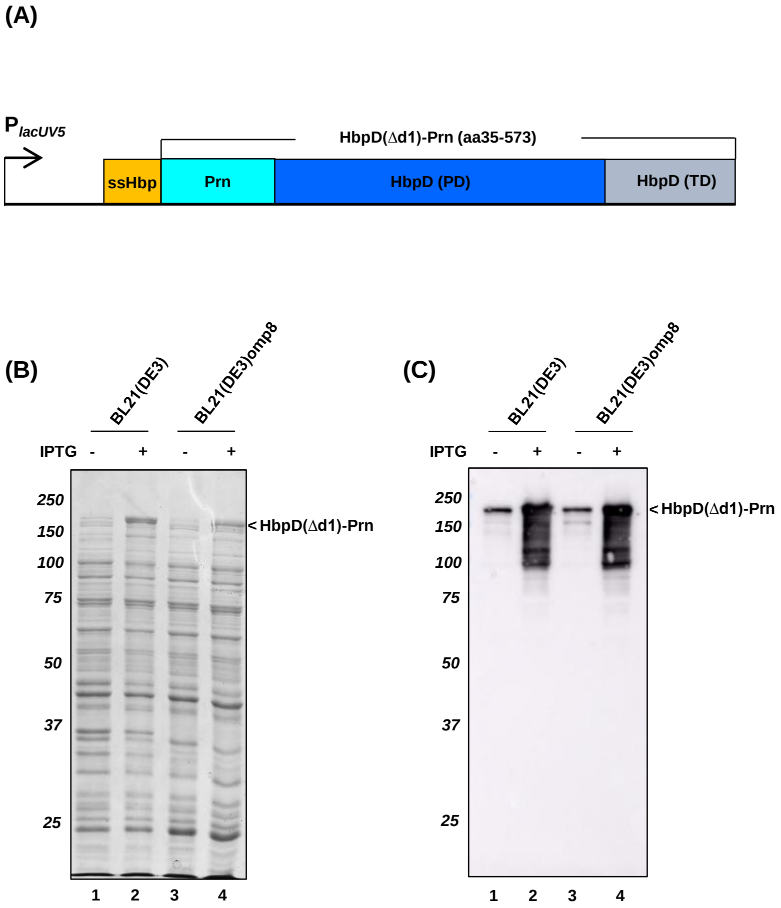

Fig. S3: Expression of HbpD(Δd1)-Prn in different *E. coli* strains. (**A**) Schematic representation of pEH3-HbpD(Δd1)-Prn (aa35-573) construct in which truncated Prn (aa35-573) was replaced for domain 1 (Δd1) in the HbpD(Δd1)-based platform under control of the *lacUV5* promoter and the signal sequence of *E. coli* Hbp (ssHbp) was fused to the fusion of HbpD(Δd1)-Prn at its N-terminus. (**B** and **C**) BL21(DE3) and BL21(DE3)omp8 cells were induced for HbpD(Δd1)-Prn expression with (+) or without (-) 0.1 mM IPTG for 2 h and whole cells were analyzed by SDS-PAGE followed by Coomassie staining and immunoblotting using antiserum against Hbp. MW (kDa) markers were indicated at the left side of panels, the identified protein bands were indicated at the right side of panels.

Fig. S4

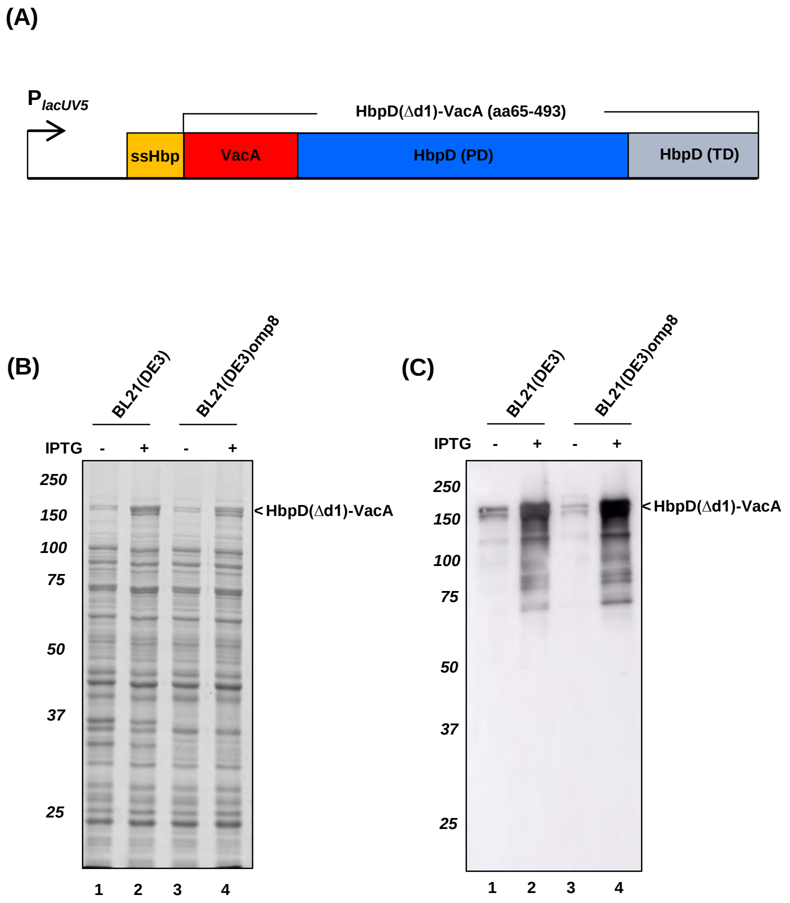

Fig. S4: Expression of HbpD(Δd1)-VacA in different *E. coli* strains. (**A**) Schematic representation of pEH3-HbpD(Δd1)-VacA (aa65-493) construct in which truncated VacA (aa65-493) was replaced for domain 1 (Δd1) in the HbpD(Δd1)-based platform under control of the *lacUV5* promoter and the signal sequence of *E. coli* Hbp (ssHbp) was fused to the fusion of HbpD(Δd1)-VacA at its N-terminus. (**B** and **C**) BL21(DE3) and BL21(DE3)omp8 cells were induced for HbpD(Δd1)-VacA expression with (+) or without (-) 0.1 mM IPTG (2 h) and whole cells were analyzed by SDS-PAGE followed by Coomassie staining and immunoblotting using antiserum against Hbp. MW (kDa) markers were indicated at the left side of panels, the identified protein bands were indicated at the right side of panels.

Fig. S5

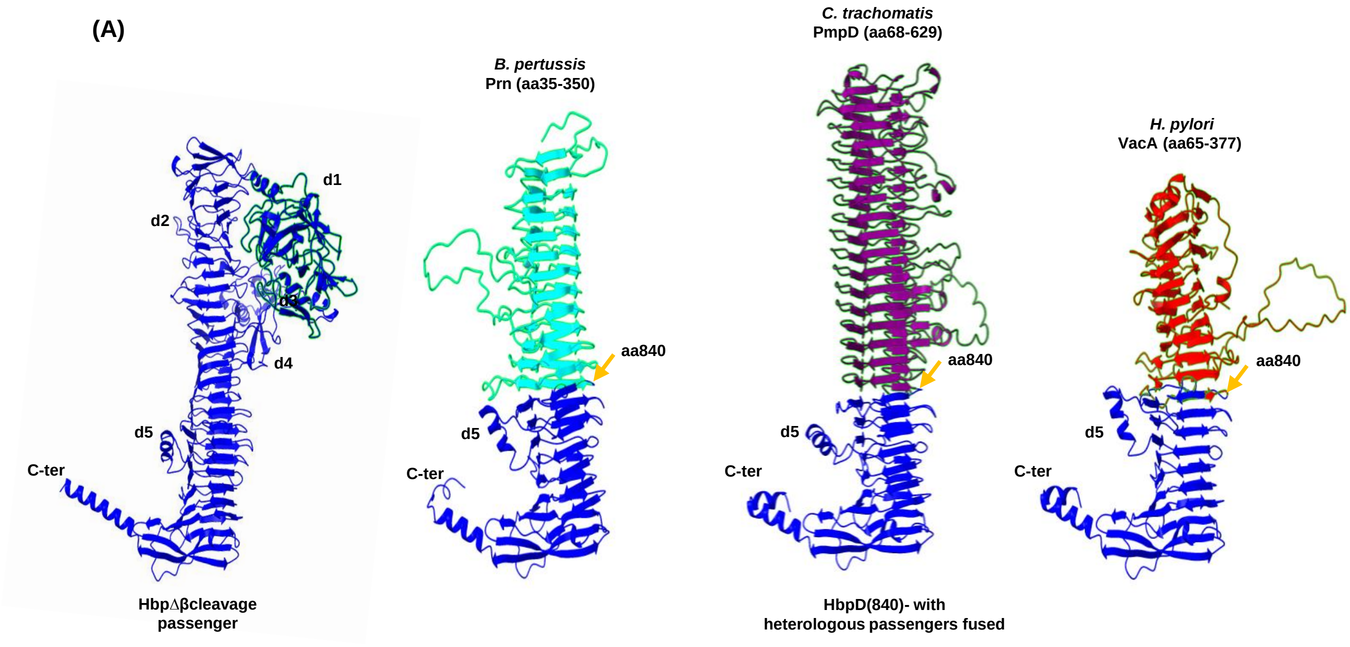

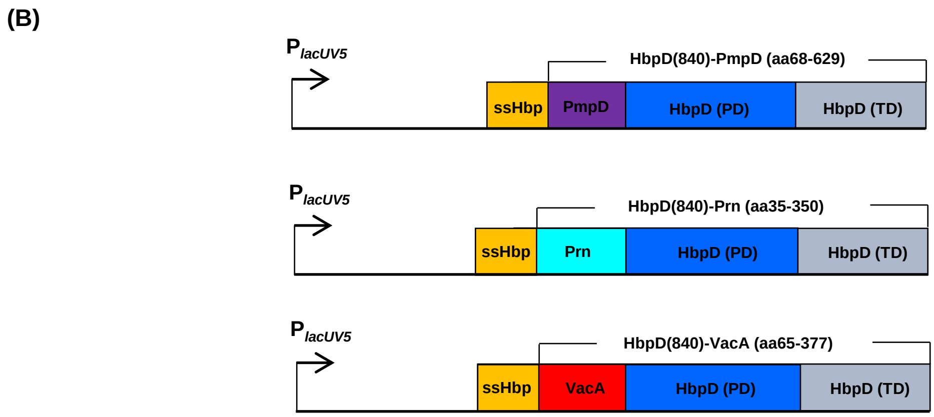

Fig. S5: Structural models and constructions of heterologous HbpD(840)-fusions. (**A**) Cartoon representation of conformations predicted by Alfafold 2 of HbpΔβcleavage, containing a passenger with five side-domains d1-d5 and of three expressed versions of HbpD(840) constructs with heterologous passenger fragments fused: HbpD(840)-PmpD (aa68-629), HbpD(840)-Prn (aa35-350) and HbpD(840)-VacA (aa65-377). The fused partner was linked at the position of residue 840 (arrow) in the Hbp passenger. (**B**) Schematic representation of the constructs in pEH3-HbpD(840)-PmpD (aa68-629), pEH3-HbpD(840)-Prn (aa35-350) and pEH3-HbpD(840)-VacA (aa65-377). Sequences encoding truncated passenger fragments of PmpD (aa68-629), Prn (aa35-350) and VacA (aa65-377) were included in between those encoding the Hbp signal sequence (ssHbp) and HbpD(840) and resulted in passengers as modelled in (A).
